# Meniscus extrusion is a predisposing factor for determining arthroscopic treatments in partial medial meniscus posterior root tears

**DOI:** 10.1186/s43019-023-00182-6

**Published:** 2023-03-14

**Authors:** Takayuki Furumatsu, Keisuke Kintaka, Naohiro Higashihara, Masanori Tamura, Koki Kawada, Haowei Xue, Toshifumi Ozaki

**Affiliations:** Department of Orthopaedic Surgery, Okayama University Graduate School of Medicine, Dentistry, and Pharmaceutical Sciences, 2-5-1 Shikatacho, Kitaku, Okayama 700-8558 Japan

**Keywords:** Medial meniscus, Posterior root, Partial tear, Meniscal extrusion, Operative indication

## Abstract

**Background:**

Patients with partial medial meniscus posterior root tears (MMPRTs) sometimes require arthroscopic pullout repair because of their intolerable/repeated knee pains and continuous disturbance in gait during activities of daily living. However, the predisposing factors for future knee surgery in patients with partial MMPRTs remain unclear. We compared the findings of magnetic resonance imaging (MRI) between patients who underwent pullout repair and nonoperative management following partial MMPRTs.

**Methods:**

Twenty-five patients who required arthroscopic repair for partial MMPRTs and 23 patients who were managed nonoperatively were evaluated during a mean follow-up period of 27.1 months. Sex, age, height, body weight, body mass index, duration from onset to initial MRI, MRI findings, and medial meniscus (MM) extrusion were compared between the two groups. Linear regression analysis was used to assess the correlation between MM extrusion and duration from onset to MRI examination.

**Results:**

No significant differences were observed between the pullout repair and nonoperative management groups in terms of patient demographics and the positive ratio of MRI-based root tear signs. However, absolute MM extrusion in the pullout repair group (3.49 ± 0.82 mm) was larger than that in the nonoperative management group (2.48 ± 0.60 mm, *P* < 0.001). Extrusion of the MM (> 3 mm) was detected more frequently in the pullout repair group than in the nonoperative management group (*P* < 0.001). The odds ratio in the pullout repair and MM extrusion > 3 mm cases was 9.662. Linear regression analysis revealed a fair correlation between the duration from onset to MRI and MM extrusion only in the pullout repair group (0.462 mm/month increase in MM extrusion).

**Conclusions:**

This study demonstrated that more severe MM extrusions were observed in the pullout repair group than in the nonoperative management group. Major extrusion (> 3 mm) was also observed more in the pullout repair group than in the nonoperative group. Assessing MM extrusion and its severity can help determine a valid treatment for patients with partial MMPRTs.

**Level of evidence:**

IV, Retrospective comparative study.

## Introduction

A medial meniscus (MM) posterior root can serve as an essential anchor to stabilize the MM during complicated knee motion. MM posterior root tears (MMPRTs) are known to cause serious meniscus damage that induces MM extrusion, progressive cartilage loss, osteoarthritis, and subchondral insufficiency fracture of the knee (SIFK) by disrupting MM function [1]. Partial MMPRTs may increase the possibility of developing complete tears of the MM posterior root in the case of nonoperative management [2, 3]. However, the predisposing factors for determining future arthroscopic treatments in patients with partial MMPRTs during the follow-up period have remained unclear.

In magnetic resonance imaging (MRI) analyses, MM posterior root lesions include three types of MRI-based appearances: degeneration, characterized by thickening of the root with intrasubstance hyperintensity not contacting the articular surface; partial tear, characterized by abnormal signal intensity extending to the articular surface or abnormal root morphology with partial root discontinuity; and complete tear, characterized by complete discontinuity of the affected root [4]. A partial radial tear of the MM posterior root (LaPrade arthroscopic/morphological classification type 1 [5]) shows fluid signal intensity at the root insertion and subchondral and/or subenthesial linear bone marrow signal intensity on MRIs [6]. Furumatsu et al. demonstrated that an ocarina-like appearance with several condensed circles in the triangular meniscal horn (ocarina sign) is the most common MRI finding in patients with partial MMPRTs [7]. Partial MMPRTs sometimes require arthroscopic meniscus repairs because of intolerable/repeated knee pain, even though they have not progressed to complete tears of the MM posterior roots.

This study aimed to compare MRI findings between the pullout repair and nonoperative management groups following partial MMPRTs. We hypothesized that a larger MM extrusion would be observed on preoperative MRI in the pullout repair group than in the nonoperative management group following partial MMPRTs.

## Patients and methods

**Fig. 1:**
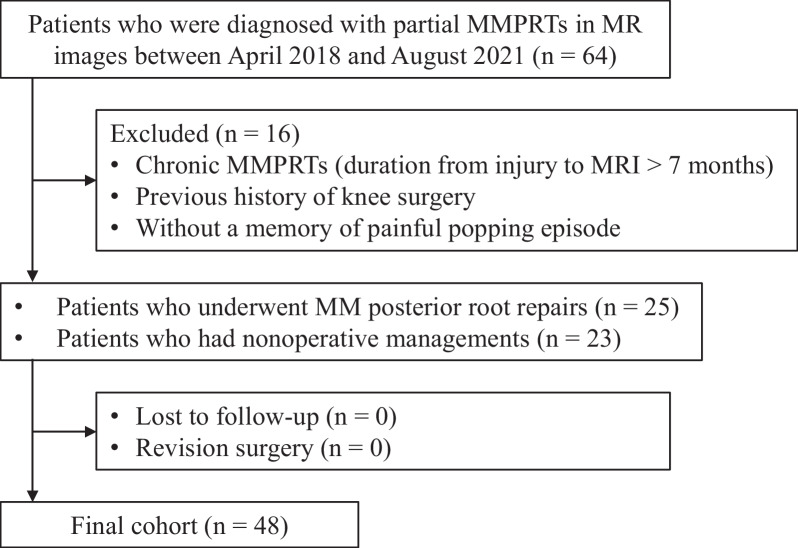
Flow diagram illustrating the selection of patients in this study

This study received the approval of our institutional review board and written informed consent was obtained from all patients. Sixty-four patients who were diagnosed with partial MMPRTs using MRIs [7] between April 2018 and August 2021 were evaluated (Fig. 1). The estimated statistical power was 0.979 using each sample size of 32 (difference of mean MM extrusion between two groups, 1 mm; standard deviation, 1 mm; α error, 0.05). Patients were diagnosed as having partial MMPRTs if they had two or more positive MRI findings of the following: root irregularity, bone marrow spot (sagittal or coronal images), and ocarina sign [7]. Patients diagnosed with complete MMPRTs were not included. Patients who had chronic MMPRTs at the first MRI examination (duration from onset to MRI > 7 months) were excluded (*n* = 3). Patients who had a previous history of knee surgery (*n* = 2) and patients without a concrete memory of posteromedial painful popping episode (*n* = 11) were also excluded. Memory of injury patterns and the date of sudden posteromedial painful popping of the knee, characteristic episodes of patients with MMPRTs, were obtained from the patients through careful interviews [8, 9]. MMPRTs are frequently occurred during descending knee motion such as stair descent and going downhill, with an episode of posteromedial painful popping [9]. We determined the date of MMPRT onset by the interviews. Twenty-five patients required arthroscopic pullout repairs of the MM posterior root because of symptomatic knee pains and knee dysfunction during the follow-up period. All patients who underwent pullout repairs showed partial MMPRTs. Patients who refused arthroscopic surgery were included in the nonoperative management group. There were no patients who underwent surgery to prevent the progression of knee osteoarthritis even though they had no severe pain. The remaining 23 patients were managed nonoperatively. We compared these two groups retrospectively as a final cohort (*n* = 48). Patient demographics are presented in Table 1.

**Table 1:** Patient demographics

	Pullout repairs	Nonoperative managements	*P*-value
Number of patients	25	23	
Gender, men/women	10/15	4/19	0.117^a^
Age in years (range)	66.6 ± 9.5 (47–85)	62.2 ± 10.4 (39–84)	0.069
Height in m (range)	1.59 ± 0.08 (1.43–1.78)	1.60 ± 0.05 (1.49–1.73)	0.293
Body weight in kg (range)	67.8 ± 10.4 (49–93)	65.3 ± 19.2 (40–120)	0.318
Body mass index (kg/m^2^)	26.0 ± 3.6	26.7 ± 6.1	0.380
Femorotibial angle (°)	176.7 ± 1.0	177.0 ± 2.6	0.425
MRI examinations	35	38	
Duration from onset to initial MRI in days (range)	32.1 ± 38.9 (1–170)	41.8 ± 55.1 (1–190)	0.243
Duration from onset to surgery in days (range)	89.9 ± 66.0 (36–279)		
Partial MMPRT classification			
Type A/B/C	3/13/9		
Duration from onset to final follow-up, in months	26.3 ± 9.1	27.9 ± 12.4	0.364

Data of age, height, body weight, body mass index, femorotibial angle, and durations are displayed as a mean ± standard deviation. MRI, magnetic resonance imaging; MMPRT, medial meniscus posterior root tear; MM, medial meniscus. Statistical differences in age, height, body weight, body mass index, femorotibial angle, and durations between two groups were analyzed using a Mann–Whitney *U* test. ^a^Fisher’s exact test

### MRI examinations

**Fig. 2:**
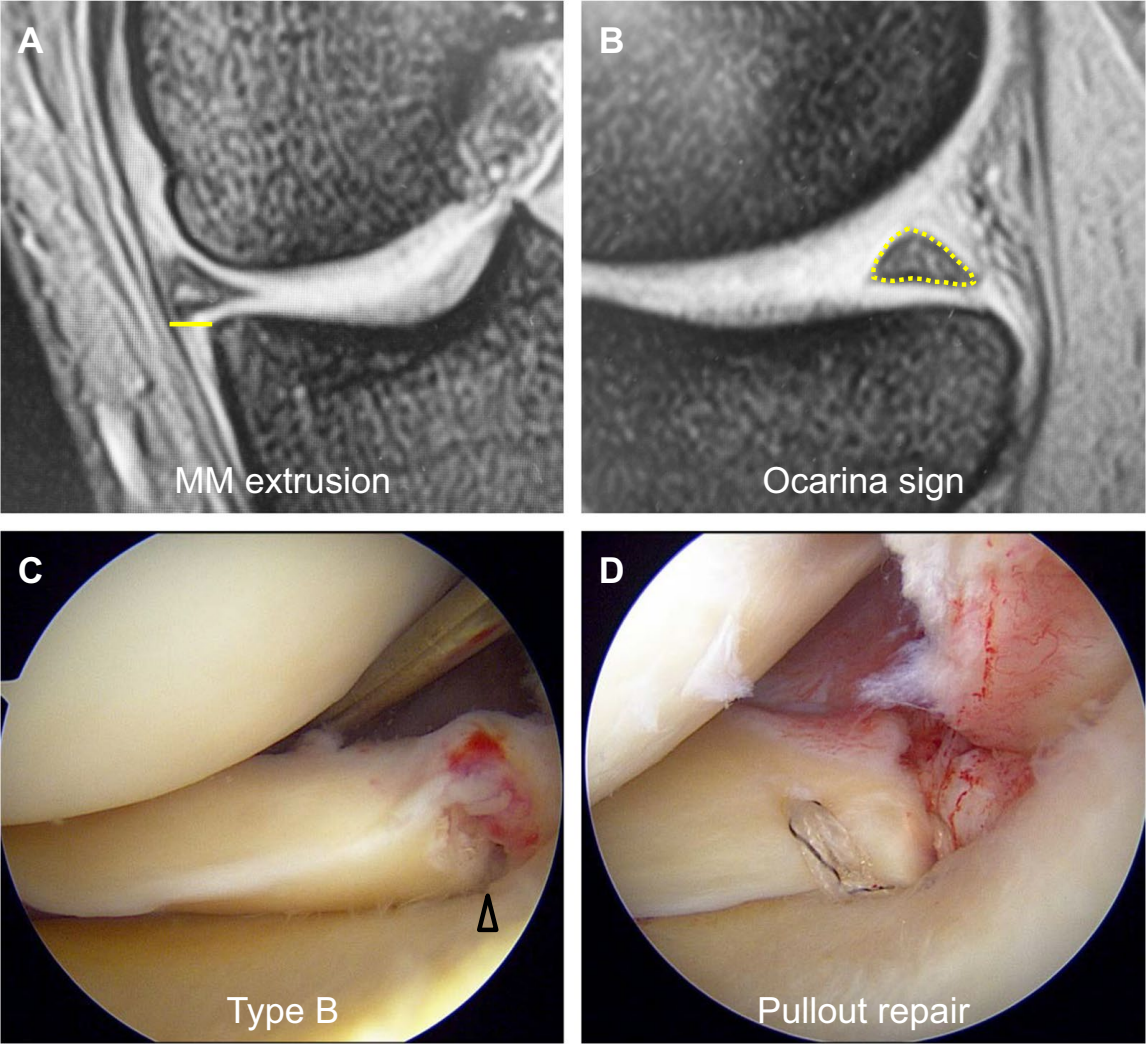
Partial MMPRT. **A** An absolute MM extrusion (yellow line). **B** An ocarina sign. Note a multiple fiber bundle formation showing several condensed circles in triangular meniscal horn (yellow-dotted area). **C** Type B partial tear (arrowhead). **D** Transtibial pullout repair

Patients were examined by preoperative MRI scans (one to two times). The overall mean duration from onset to initial MRI scan was 36.6 days. The number of MRI examinations were 35 and 38 in the pullout repair and nonoperative management groups, respectively. Multiple MRI examinations were performed in 10 of 25 pullout repair patients and 15 of 23 nonoperatively managed patients. Second MRI scans were sometimes required in patients with pain progression and/or patients who wanted reassessment in our institute. We evaluated 48 scans of the first MRIs and 25 additional scans of the second MRIs. MRI scans were mainly obtained using an Achieva 1.5 T (Philips, Amsterdam, the Netherlands) or an EXCELART Vantage Powered by Atlas 1.5 T (Toshiba Medical Systems, Otawara, Japan) with a knee coil. Standard sequences of the Achieva included sagittal [repetition time (TR)/echo time (TE) 742/18], coronal (TR/TE 637/18), and axial (TR/TE 499/18) T2-weighted fast-field echo with a 20° flip angle (FA). Standard sequences of the Vantage included sagittal and coronal proton density (PD) fast-spin-echo (TR/TE 2300/18), and axial T2-weighted fat suppression (TR/TE 3500/60) with a 90° FA. Slice thickness was 3 mm with a 0.6-mm gap. Field of view (FOV) was 16 (or 17) cm with an acquisition matrix size of 205 × 256 (or 200 × 368) [10]. Coronal images were obtained along with a section parallel to a tangential line between both posterior femoral condyles. Sagittal images were set perpendicular to the coronal images. Axial images were obtained according to the position of both menisci. Conventional MRI-based findings of complete MMPRTs such as cleft, giraffe neck, medial extrusion, ghost, and radial tear signs [10] were evaluated. Medial extrusion of the MM was measured from the medial margin of the tibial plateau to the outer border of the MM on the coronal image that crossed the midpoint of the anteroposterior length of the MM (Fig. 2A). MM extrusion > 3 mm was defined as a progressive/pathological extrusion sign [4, 6, 10, 11]. Characteristic MRI findings of partial MMPRTs were also assessed (ocarina sign [Fig. 2B], root irregularity, and bone marrow spot [7, 11]). No patients were diagnosed as having sequential complete MMPRTs in MRIs during the follow-up.

### Surgical indications

Patients having partial MMPRTs in MRI examinations were treated with nonoperative management initially. Nonoperative management included deep-knee-bend prevention, prohibition of squatting knee motion, one step at a time stair-climbing, sideways or backward stair-descending [12], walking with a cane as tolerated, avoidance of carrying heavy luggage, prescription of analgesic medications as needed, and weight control [13]. Prolonged night pain of the knee and/or continuity of a sleeper’s sign, defined as nighttime medial tibiofemoral pain when the patient is in the fetal position with both knees in contact [14] were often observed. If a patient’s pain persisted with increased functional disability at 6 weeks following the painful popping event, the patient was regarded as having a clinical failure of the nonoperative management and we suggested surgical treatments as the second step to the patient. However, a strong demand for surgical treatment was accepted without waiting for 6 weeks in patients who could not tolerate severe knee pain. All patients met the following items for isolated MM posterior root repairs in our institute: femorotibial angle ≤ 180°, Kellgren-Lawrence grades ≤ 2, SIFK grade ≤ 2 [15], and no severe cartilage loss in the medial compartment. All patients in both groups met the radiographic/MRI-based surgical indications. In the surgically treated group, patients received arthroscopic pullout repairs of the MM posterior root using two cinch stitches or two simple stitches combined with a posteromedial pullout suture (Fig. 2). We performed transtibial pullout repairs using two nonabsorbable sutures (No. 2 Ultrabraid, Smith and Nephew, Andover, MA, USA) with an all-inside meniscal repair device (FAST-FIX, Smith and Nephew). A Knee Scorpion suture passer (Arthrex, Naples, FL, USA) was used for passing the sutures. A tibial tunnel was created at the anatomical attachment of the MM posterior root using a root aiming guide, 2.4 mm guide wire, and 4.0 mm cannulated reamer (Arthrex). In the postoperative rehabilitation, patients were kept non-weight bearing for 2 weeks. Between 2 and 4 weeks, knee flexion exercise was increased to 30°, 60°, and 90° under partial weight-bearing conditions (20, 40, and 60 kg). After 5 weeks, the patients were allowed full weight bearing and knee flexion > 120°. If a patient’s pain was improved and functional disability was decreased after 6 weeks of nonoperative management, the patient was consistently followed-up with activity modifications involved in deep-knee-bend prevention and weight control. There was no difference in the above conditions between the two groups except for symptom improvement after conservative treatment.

### Partial MMPRT classification

Following the medial joint space-widening procedure (the outside-in pie-crusting technique [16]), partial MMPRTs were determined by careful arthroscopic examinations according to partial MMPRT classification [7]. A partial tear/damage of the MM posterior root was defined as an incomplete structural cleavage between 0 and 9 mm from the native MM posterior root attachment. According to the arthroscopic classification of partial MMPRTs [7], a partial tear/damage of the MM posterior root was divided into the following three types: type A, accurate partial stable tear (cleavage < 1/2 of root width); type B, bridged unstable root tear (cleavage ≥ 1/2 of root width, Fig. 2C); and type C, complex horn tear expanded to the root. A completely detached root with a gap (complete MMPRT) was not included in this study. Sequential complete MMPRTs following MRI-based partial MMPRTs were not observed at arthroscopic evaluations.

### Statistical analysis

Data were presented as means ± standard deviations. Statistical differences in age, height, body weight, body mass index, durations, and absolute MM extrusion between two groups were analyzed using a Mann–Whitney *U*-test. Differences in gender and MRI signs between groups were compared using Fisher’s exact test. Power and statistical analyses were performed using EZR (Saitama Medical Center, Saitama, Japan), which is a graphical user interface for R (the R Foundation for Statistical Computing). Significance was set to *P* < 0.05. A sample size calculation was performed with a significance level of 0.05 and a power of 0.80. The resulting requirement for sample size in MM extrusion was 12 in both groups. Linear regression analysis was used to assess the correlation between absolute MM extrusion and duration from onset to MRI examination. All MRI measurements and duration from onset to MRI examination was used in the linear regression analysis. A good correlation was represented by *R*^2^ ≥ 0.60, fair correlation by *R*^2^ ≥ 0.50, and poor correlation by *R*^2^ < 0.50. Two orthopedic surgeons independently assessed MRIs in a blinded manner. Each observer performed each evaluation twice, at least 2 weeks apart. The reliability of MRI evaluation was assessed by examining the interobserver and intraobserver reliabilities. The interobserver and intraobserver reliabilities were assessed using an intraclass correlation coefficient (ICC). An ICC > 0.80 was considered to represent a reliable measurement.

## Results

No significant differences were observed between the pullout repair and nonoperative management groups in terms of patient demographics (Table 1). Among patients who required pullout repairs during follow-up, partial MMPRTs were observed (type A, 3 knees; type B, 13 knees; type C, 9 knees). No complete MMPRTs were observed in pullout repair cases. The mean duration from the popping event to surgery was 89.9 ± 66.0 days (range 36–279 days). The mean follow-up duration was 27.1 ± 10.7 months, with a minimum follow-up duration of 12 months.

**Fig. 3:**
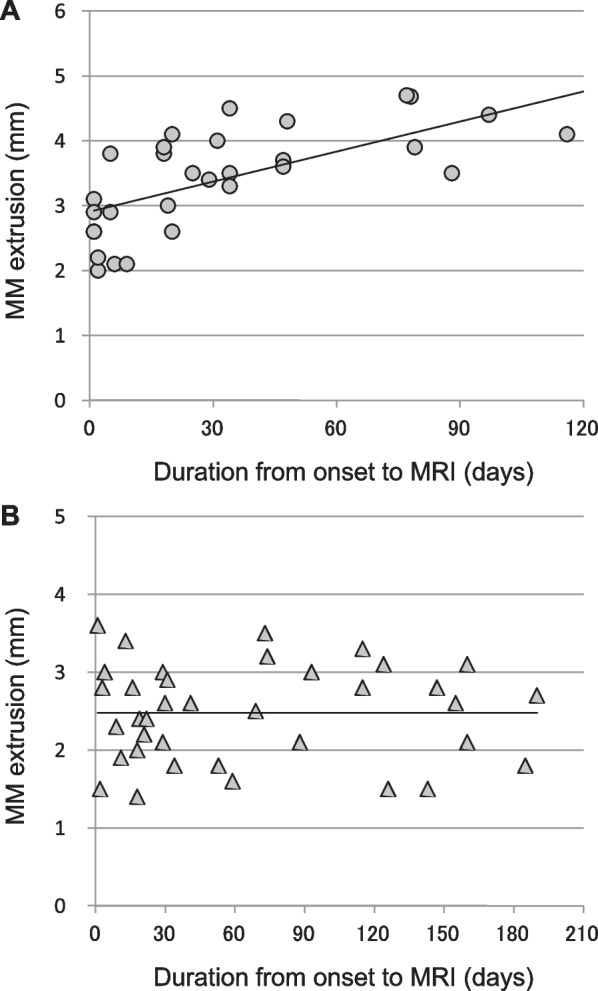
Correlation between duration from onset to MRI examination and MM extrusion. **A** In the pullout repair group, the regression equation was linear: MM extrusion = 0.015 × duration + 2.908 mm (*R*^2^ = 0.54, *P* < 0.001, 0.462 mm/month increase). **B** No significant correlation between duration and MM extrusion was observed in the nonoperative management group

**Fig. 4:**
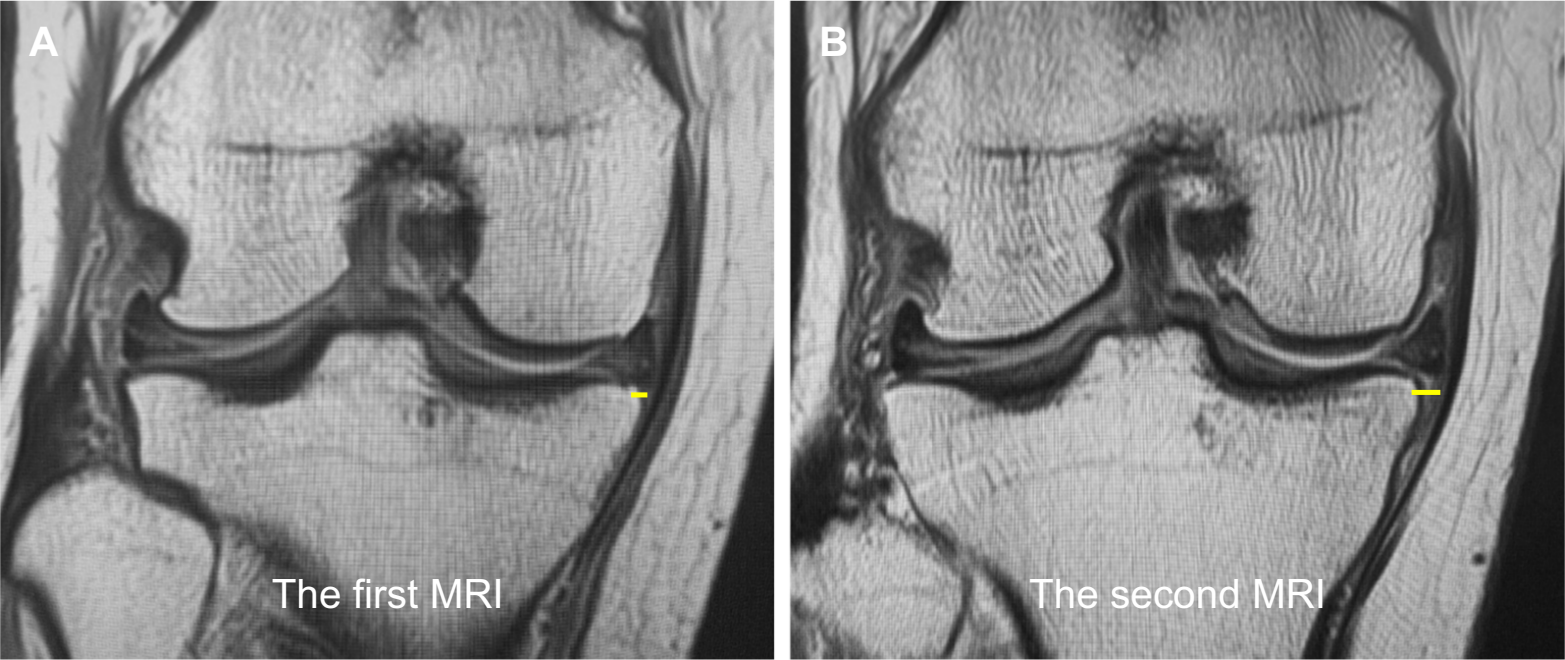
MM extrusion. A 62-year-old female patient in the pullout repair group. **A** Finding from the first MRI examination performed 5 days after the painful popping event. **B** A second MRI examination was performed 47 days after the onset of partial MMPRT. Yellow lines indicate MM extrusions

The pullout repair and nonoperative management groups underwent 35 and 38 MRI examinations, respectively. The characteristic MRI findings of partial and/or complete MMPRTs were evaluated. No significant differences were observed between the two groups in terms of the positive ratio of the cleft, giraffe neck, ghost, ocarina, radial tear signs, root irregularity, or bone marrow spot (Table 2). However, absolute MM extrusion in the group that required pullout repair (3.49 ± 0.82 mm) was larger than that in the nonoperative management group (2.48 ± 0.60 mm, *P* < 0.001). Furthermore, extrusion of the MM (> 3 mm) was detected more frequently in the pullout repair group (68.6%) than in the surgery-free nonoperative management group (18.4%, *P* < 0.001). The odds ratio involved in pullout repair and MM extrusion > 3 mm was 9.662. Linear regression analysis showed a fair correlation between the duration from onset to MRI examination and MM extrusion in the pullout repair group [MM extrusion = 0.015 × disease duration (days) + 2.908 mm, *R*^2^ = 0.54, *P* < 0.001, Fig. 3A]. Using the above formula, MM extrusion showed a 0.462 mm/month increase in patients requiring pullout repair. In contrast, no significant correlation was observed between duration and MM extrusion in the nonoperative management group (Fig. 3B). In the pullout repair group, a 0.80 ± 0.59 mm increase was observed in the absolute MM extrusion during a mean of 62.4 days between the first and second MRI scans (*n* = 10). In the nonoperative management group, a 0.19 ± 0.60 mm increase in MM extrusion was observed during a mean of 77.9 days between the first and second MRI scans (*n* = 15). A preoperative time-dependent increase in MM extrusion was observed in the pullout repair group (Fig. 4). Following pullout repairs, MM extrusion was 3.80 ± 0.64 mm at 3 months postoperatively. The interobserver reproducibility and intraobserver repeatability of the MRI findings were satisfactory, with mean ICC values of 0.83 and 0.86, respectively.

**Table 2:** Characteristic MRI findings in MMPRTs

	Pullout repairs (*n* = 35)	Nonoperative managements (*n* = 38)	*P*-value
Coronal images			
Cleft sign (%)	8 (22.9)	4 (10.5)	0.211^a^
Giraffe neck sign (%)	9 (25.7)	7 (18.4)	0.574^a^
MM extrusion (mm)	3.49 ± 0.82	2.48 ± 0.60	< 0.001*^b^
MM extrusion > 3 mm (%)	24 (68.6)	7 (18.4)	< 0.001*^a^
Root irregularity (%)	16 (45.7)	12 (31.6)	0.238^a^
Bone marrow spot (%)	21 (60.0)	21 (55.3)	0.813^a^
Sagittal images			
Ghost sign (%)	7 (20.0)	8 (21.1)	1.000^a^
Ocarina sign (%)	31 (88.6)	31 (81.6)	0.519^a^
Bone marrow spot (%)	13 (37.1)	19 (50.0)	0.346^a^
Axial images			
Radial tear sign (%)	5 (14.3)	4 (10.5)	0.723^a^

Data of medial meniscus (MM) extrusion are displayed as a mean ± standard deviation. MRI, magnetic resonance imaging; MMPRT, medial meniscus posterior root tear. ^a^Fisher’s exact test. ^b^Mann–Whitney *U* test. *Significant difference

## Discussion

The most important finding of this study was that the MM extrusions were larger in the pullout repair group than in the nonoperative management group, and major MM extrusion > 3 mm was observed frequently. Our hypothesis was also proved that patients with large MM extrusion would require surgery due to severe knee pain and dysfunction. Our results suggest that measuring absolute MM extrusion twice, 1–3 months apart, may help determine the early intervention of surgical treatments in patients with partial MMPRTs.

In asymptomatic adults without knee pain and radiographic knee osteoarthritis (mean age, 55 years), MM extrusion reaches 1.64 mm at baseline [17]. This cohort showed a slight increase in MM extrusion over 4 years (0.040 mm/year). In contrast, MM extrusion progresses shortly after MMPRT onset [18]. Absolute MM extrusion increases up to 4.2 ± 1.2 mm at 1–3 months following MMPRT onset [18]. Several authors reported that MM extrusion increases progressively within a short duration after the onset of MMPRTs in symptomatic patients who require surgical treatment of the knee (0.014–0.020 mm/day) [19, 20]. In this study, a rapid increase in MM extrusion (0.462 mm/month) was observed in partial MMPRTs that required arthroscopic pullout repair. Additionally, no complete MMPRTs were observed in the pullout repair group. No significant correlation was observed between disease duration and MM extrusion in patients with partial MMPRT who did not require surgical treatment (Fig. 3). These findings suggest that arthroscopic surgery is required owing to severe MM extrusion and knee dysfunction in patients with partial MMPRTs. We speculated that the pathophysiology of MM extrusion increasing partial MMPRTs would be similar to that of complete MMPRTs or may be a precursor of complete MMPRTs. MM extrusion may be increased by sequential progression to complete tears and worsening of knee osteoarthritis.

The status of MM extrusion is an important factor affecting the fate of MMPRTs and the progression of knee osteoarthritis [13, 21]. In MMPRT patients who require surgeries as final treatments, absolute MM extrusion is larger than in conservatively treated MMPRTs during 3.75 years of the average follow-up period (3.42 mm versus 1.36 mm) [21]. Kwak et al. describe that a large MM extrusion ratio (absolute MM extrusion/medial femoral condyle width, > 0.08) was the most reliable poor prognostic factor for the conservative treatment of MMPRTs [13]. In contrast, a lesser extent of MM extrusion (2.98 mm) was significantly associated with nonoperatively survived patients following MMPRTs [22]. These studies were primarily based on data from patients with complete MMPRTs. Our study demonstrated that patients with true partial MMPRTs who required pullout repair showed a larger MM extrusion (3.49 mm) than the nonoperatively managed surgery-free patients (2.48 mm). Therefore, we speculated that valid decision making is important at an early stage even in patients with partial MMPRTs. If a patient shows severe MM extrusion ≥ 3.5 mm at 1 month after the onset of partial MMPRT (≥ 4 mm at 2 months; calculated using the linear equation, MM extrusion = 0.015 × duration + 2.908 mm), early arthroscopic pullout repair could be speculated as the treatment option for partial MMPRT. If a patient shows slight MM extrusion and/or little progression of MM extrusion, nonoperative management could be continued as the primary treatment.

MM extrusion is a characteristic MRI finding in complete MMPRTs. However, MM extrusion is not a specific finding in complete MMPRTs [10]. In addition, the pullout repair group following partial MMPRTs showed a high positive ratio of MM extrusion of > 3 mm (68.6%, Table 2). We speculate that the deterioration of MM function may be related to severe MM extrusion. In this study, characteristic MRI findings of complete MMPRTs (cleft, giraffe neck, and ghost signs) were also observed in partial MMPRTs (Table 2). These signs are observed in complete MMPRTs [10]. However, the positive ratio of these signs is low in partial MMPRTs [7]. Three types of partial MMPRTs have been reported: type A, accurate partial stable tear (cleavage < 1/2 of root width); type B, bridged unstable root tear (cleavage ≥ 1/2 of root width); and type C, complex horn tear expanded to the root [7]. In type B partial MMPRTs, the anterior gap of the root may be detected as cleft and/or giraffe neck signs in coronal images (ghost sign in sagittal images). We consider that the cross-section setting in MR image acquisition would induce positive findings of these signs in partial MMPRTs.

Degeneration and partial tear/damage to the MM posterior root are sometimes detected on symptomatic knee MRIs [4]. The rate of partial MMPRTs accounts for 40.8% of MRI-diagnosed MM posterior root ligament lesions despite a lower rate of complete MMPRTs (9.2%) [4]. On the other hand, several authors have demonstrated that the rate of type 1 partial MMPRTs accounts for 3.9–16.4% in patients with MMPRTs who underwent arthroscopic treatments [23–26]. Based on these findings, arthroscopic treatment is not often required in most patients diagnosed with partial MMPRTs using MRI examinations. Otherwise, many partial MMPRTs may remain unnoticed in clinical examinations and MRI assessments. In our study, MRI-diagnosed partial MMPRTs were confirmed arthroscopically as true partial MMPRTs in all patients who underwent pullout repairs. We speculated that an accurate MRI-based diagnosis of partial MMPRTs using several characteristic MRI findings [7] and MM extrusion is important for identifying cases that may require arthroscopic pullout repair.

This study had several limitations. This was a retrospective comparative study that included a small number of patients. The date of MMPRT onset was determined through patient interviews. The follow-up period in this study was relatively short. MR images were not perfectly obtained in the same situation (different number of examinations and different durations from onset to MRI scans). Multiple MRI scans were not performed for every patient. It is possible that the appearance of characteristic MRI findings may depend on the duration between onset and MRI examination [27]. Partial MMPRTs in the nonoperative management group were not confirmed arthroscopically. Repeated MRI examinations in the same patients are required to accurately evaluate the time-dependent progression of MM extrusion in patients with partial MMPRTs.

## Conclusions

This study demonstrated that MM extrusions (> 3 mm) were observed more frequently in the pullout repair group than in the nonoperative management group following partial MMPRTs. Major MM extrusion (> 3 mm) was also a predisposing factor for determining arthroscopic pullout repairs in partial MMPRTs. Assessing MM extrusion and its severity can help determine a valid treatment for patients with partial MMPRTs.

## Data Availability

Not applicable.

## Abbreviations

MM: Medial meniscus
MMPRT: Medial meniscus posterior root tear
MRI: Magnetic resonance imaging
